# Clinical features and outcomes of diffuse endocapillary proliferation Henoch-Schönlein purpura nephritis in children

**DOI:** 10.6061/clinics/2016(09)11

**Published:** 2016-09

**Authors:** Haidong Fu, Jianhua Mao, Yanping Xu, Weizhong Gu, Xiujuan Zhu, Aimin Liu

**Affiliations:** The Children’s Hospital of Zhejiang University School of Medicine, Department of Nephrology, Hangzhou 310003, China

**Keywords:** Henoch-Schönlein Purpura Nephritis, Diffuse Endocapillary Proliferation, Renal Biopsy

## Abstract

**OBJECTIVE:**

To investigate the outcomes of childhood diffuse endocapillary proliferation Henoch-Schönlein purpura nephritis (DEP-HSPN) in response to early diagnosis and prompt treatment.

**METHODS:**

Eleven cases of DEP-HSPN in children were investigated in comparison to HSPN without diffuse endocapillary proliferation (non-DEP-HSPN).

**RESULTS:**

DEP-HSPN had a higher prevalence of nephrotic syndrome but a lower prevalence of hematuria compared to non-DEP-HSPN. IgA, IgG and IgM antibody deposition was found in DEP-HSPN by histopathological examination. Proteinuria cleared in all 11 cases through treatment with steroids and/or immunosuppressive drugs. However, half of the DEP-HSPN patients continuously had hematuria after treatment.

**CONCLUSION:**

The early diagnosis and prompt initiation of immunosuppressive treatment based on renal biopsy are important for achieving favorable outcomes.

## INTRODUCTION

Henoch-Schönlein purpura (HSP) is an immunoglobulin A (IgA)-mediated, systemic small-vessel vasculitis that occurs in children 1. The deposition of IgA antibodies in vessel walls leads to symptoms involving the skin, joints, intestines and kidneys 2. Henoch-Schönlein purpura nephritis (HSPN), which is defined as HSP for 6 months with kidney involvement, including hematuria and/or proteinuria, is the most serious complication and often determines the prognosis of an HSP patient 1,2.

The progression of renal involvement is important in evaluating HSPN prognosis and in selecting individualized therapeutic strategies. The clinical manifestations of HSPN are miscellaneous and the pathological features are divergent 3,4. In addition, the severity of the clinical manifestations of HSPN in children does not always correlate with the severity of the pathological renal biopsy findings 3. HSPN with diffuse endocapillary proliferation (DEP-HSPN) may have a different prognosis than HSPN without diffuse endocapillary proliferation (non-DEP-HSPN). Therefore, in the current study, the pathological features and corresponding clinical manifestations of DEP-HSPN were compared with those of non-DEP-HSPN in children.

## METHODS

### Subjects

From December 2007 to November 2013, 4212 HSP patients were hospitalized in the Children's Hospital, School of Medicine, Zhejiang University. Of these patients, 1823 were diagnosed with HSPN and 503 cases were further confirmed by renal biopsy with indications of the following diseases or manifestations: 1) nephritic syndrome; 2) moderate or severe proteinuria; 3) acute nephritis; or 4) rapidly progressive glomerulonephritis. The 503 patients were enrolled into the current study and grouped as DEP-HSPN (n=11) and non-DEP-HSPN (n=492). A written consent form was obtained from each patient’s parents. The study protocol was approved by the Ethics Committee of Zhejiang University. The diagnosis of HSP fulfills the criteria for the classification of childhood vasculitis 5. The inclusion criteria for this prospective study were as follows: 1) patients were ≤16 years old; 2) patients presented with typical palpable purpura or petechiae and at least one of the following: arthralgia or arthritis, abdominal pain or renal involvement. The following diseases were excluded: thrombocytopenia, post-streptococcus-infection glomerulonephritis, Parvovirus B19 infection, lupus nephritis, ANCA-associated nephritis, hepatitis B-induced nephritis, and coagulopathy. HSPN was defined as the presence of gross or microscopic hematuria (>5 erythrocytes per high-power field) with or without proteinuria (>4 mg/kg/d), nephrotic syndrome (urine protein >50 mg/kg/d, serum albumin <2.5 g/dL, edema and hyperlipidemia), or acute nephritis (hematuria plus at least one of the following: increased serum creatinine, hypertension, or oliguria).

### Histopathology

Kidney biopsies were obtained from all 503 patients and were stained with hematoxylin and eosin (H&E) and for immunofluorescence (IF). IF staining was performed on 3-µm cryostat sections using polyclonal fluorescein-isothiocyanate-conjugated antibodies to IgG, IgA, IgM, C3, C4, C1q and fibrinogen according to the manufacturer’s instructions (DakoCytomation, Glostrup, Denmark). The intensity of IF staining was subjectively scored on scale of 0-3. The International Study of Kidney Disease in Children classification was used to classify HSPN 6.

### Statistical analysis

Chi-squared tests or student’s two-tailed *t* tests were performed. *P* values less than 0.05 were considered significant. All data analysis was carried out using the SPSS software for windows (version 13.0; SPSS, Inc., Chicago, IL).

## RESULTS

**Figure 1:**
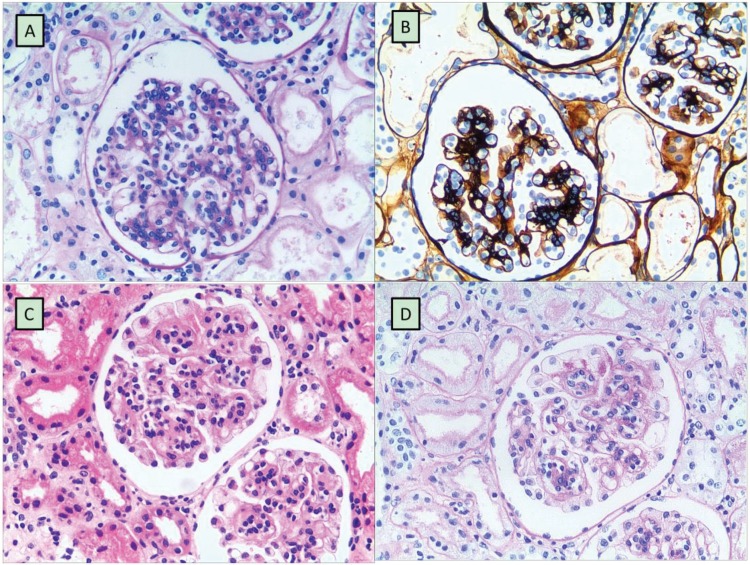
Histopathology of kidney biopsies. Figure 1A and 1B: A typical representation of DEP-HSPN characterized by significant endothelial proliferation. Figure 1C and 1D: A typical representation of non-DEP-HSPN characterized by significant mesangial cell proliferation. Biopsy samples were prepared by routine H&E staining (Figure 1A and 1C) or PAS staining (Figure 1B and 1D). Magnification = x200.

A total of 11 (4 girls and 7 boys) out of the 503 HSPN patients were given a confirmative diagnosis of DEP-HSPN, and the remaining 492 patients were diagnosed with non-DEP-HSPN. All DEP-HSPN patients had typical manifestations of HSP during the clinical visit, including skin rash, abdominal pain and joint symptoms. As shown in Table 1, of the 11 patients, 36.36% (4/11) had edema, 45.45% (5/11) had hypertension, 27.27% had gross hematuria, 72.73% had severe proteinuria (≧50 mg/kg/d), 18.18% (2/11) had moderate proteinuria (≧25 mg/kg/d, but <50 mg/kg/d), 9.09% (1/11) had mild proteinuria (<25 mg/kg/d), 27.27% (3/11) had albumin deficiency, and 9.09% (1/11) had acute renal dysfunction. The diagnosis of DEP-HSPN was pathologically confirmed by kidney biopsy in all 11 patients, and diffuse endocapillary proliferation was easily observed in the cases of DEP-HSPN via H&E staining (Figure 1A) and periodic acid-Schiff (PAS) staining (Figure 1B). In contrast, non-DEP-HSPN was characterized by the significant proliferation of mesangial cells, as indicated by H&E staining (Figure 1C) and PAS staining (Figure 1D).

As shown in Table 2, crescent formation was found in 2 of the 11 specimens and affected an average of 1.06% glomeruli (range: 0-7.69%). The clinical impact of crescent formation was not analyzed due to the limited number of cases. Of the 11 cases of DEP-HSPN, 9 were class IIb and 2 were class IIIb.

The IF staining indicated that 3 patients (27.27%) were positive for IgA, 4 cases (36.36%) were positive for IgA and IgG, 2 cases (18.18%) were positive for IgA and IgM, and 2 cases (18.18%) were positive for IgA, IgM, and IgG (Table 2). In addition, C3 deposits were found in 10 out the 11 patients (90.90%) (Table 2).

Compared to non-DEP-HSPN at the IIb stage (43 cases), DEP-HSPN (9 cases) had a higher prevalence of nephrotic syndrome (32.6% of non-DEP-HSPN *vs* 77.8% of DEP-HSPN, *p*=0.012) but a lower prevalence of hematuria (60.5% of non-DEP-HSPN *vs* 11.1% of DEP-HSPN, *p*=0.007, Table 3).

Of the 11 DEP-HSPN patients, 3 patients received methylprednisolone pulse therapy followed by prednisone and cyclophosphamide (CTX), 2 patients received prednisone plus mycophenolate mofetil (MMF), 3 patients were treated with prednisone plus Tripterygium, 2 patients were treated only with Tripterygium, and one patient was treated only with prednisone. In addition, all 11 patients were given angiotensin-converting enzyme inhibitors. As shown in Table 4, 6 patients still had hematuria after 13-20 months of treatment with MMF alone (3 cases), prednisone alone (1 case), Tripterygium alone (1 case), or methylprednisolone, prednisone, and CTX (1 case). The remaining 5 patients’ urine test results were normal after 7-17 months of treatment with Tripterygium alone (3 cases) or methylprednisolone, prednisone, and CTX (2 cases).

## DISCUSSION

The histopathological feature of HSP is the deposition of immune complexes on organs such as the skin and glomeruli 7. Glomerular nephritis in HSP patients, known as HSPN, occurs in approximately 33% of pediatric cases and approximately 63% of adult cases 8. The current study reviewed 11 cases of DEP-HSPN and 492 cases of non-DEP-HSPN. Compared to non-DEP-HSPN, DEP-HSPN had a higher prevalence of nephrotic syndrome and IgA, IgG and IgM antibody deposition but a lower prevalence of hematuria. After pulse steroid therapy followed by standard therapy with steroids with or without immunosuppressive drugs, proteinuria disappeared in all 11 cases. However, half of the DEP-HSPN patients continuously had hematuria, suggesting that hematuria in DEP-HSPN requires a more effective treatment and a longer follow-up period.

Steroid therapy is recommended for HSP patients with severe renal damage 9,10. Patients refractory to steroids may be successfully treated with immunosuppressive agents 11,12. However, there are few reports on the findings of histopathological improvement that demonstrate the effectiveness of immunosuppressive therapy for HSPN. In this regard, while 93.9% of pediatric HSPN patients and 89.2% of adult HSPN patients achieve short-term remission 13, the long-term prognosis is not always encouraging, especially in cases of adult onset. A survival rate of 74% and a complete remission rate of only 20% over a median observation period of 15 years were reported by Sherestha et al. 14. Consistently, we found that only half of the 11 DEP-HSPN cases became negative for hematuria in response to the steroid treatment with or without immunosuppressive drugs, suggesting that a longer follow-up period is required in DEP-HSPN patients.

The degrees of crescent formation, interstitial fibrosis and diffuse endothelial cell proliferation are important in predicting HSPN prognosis 14. In this context, chronic proteinuria greater than 1 g/day and/or accompanying nephrotic syndrome predict a poor HSPN prognosis 15. Therefore, it has been recommended that the treatment plan should be determined based on the clearance of or at least a reduction in proteinuria to less than 1 g/day. In the present study, proteinuria was completely cleared in DEP-HSPN patients after treatment with a single reagent or a combination of steroids and immunosuppressive drugs, suggesting that the strategies used in the current study, especially Tripterygium alone, were effective in reducing proteinuria.

In clinical practice, renal biopsies are rarely used to evaluate therapeutic efficacy. Furthermore, reducing or discontinuing an effective therapy in a patient is often carried out without confirming whether the nephritis has improved pathologically. As a result, relapse often occurs in patients who appear to have had a remission, but there is no improvement in their renal histopathology. In this study, repeat renal biopsies were not performed in the 11 cases of DEP-HSPN to monitor renal histopathological improvements and guide the therapeutic strategy, which was one of the major limitations of the current study.

The current study describes 11 cases of DEP-HSPN in comparison to cases of non-DEP-HSPN. DEP-HSPN had a higher prevalence of nephrotic syndrome but a lower prevalence of hematuria. Histopathologically, IgA, IgG and IgM antibody deposition was found in DEP-HSPN. After standard treatment with steroids and/or immunosuppressive drugs, proteinuria cleared in all 11 cases. However, half of the DEP-HSPN patients continuously had hematuria for as long as 20 months; thus, hematuria in DEP-HSPN needs to be monitored for a longer period.

## AUTHOR CONTRIBUTIONS

Fu H and Liu A contributed to the conception and design of the study. Mao J and Xu Y contributed to the data acquisition. Gu W and Zhu X contributed to the data analysis. Fu H wrote the manuscript. All of the authors reviewed and approved the final version of the manuscript.

## Figures and Tables

**Table 1:** Clinical presentation of DEP-HSPN patients.

*ID #*	*Age (yr)*	*Sex*	*RBT (w)*	*Symptoms*	*HP*	*ED*	*GH*	*PU*	*AD*	*RD*
1	5.9	M	4					Severe		
2	13.4	M	3	Arthralgia				Mild		
3	5.4	M	6		+		+	Severe		
4	5.4	M	3					Severe		
5	10.8	F	3	Abdominal pain	+			Moderate		
6	10.6	M	9			+		Severe	+	
7	7.9	F	3			+		Severe	+	
8	12.0	F	6		+		+	Severe	+	
9	7.7	F	12	Abdominal pain, arthralgia,		+		Moderate		
10	9.1	M	3	Abdominal pain, arthralgia,	+	+	+	Severe		+
11	9.2	M	2	Arthralgia	+			Severe		

RBT: time gap between clinical diagnosis to renal biopsy confirmation (weeks); HP: hypertension; ED: edema; GH: gross hematuria; PU: proteinuria; AD: albumin deficiency; RD: renal dysfunction.

**Table 2:** Histopathological examination in DEP-HSPN patients.

ID#	Immunostaining	Stage	Crescent	Time for clearance of proteinuria (Months)
1	IgA+++, IgM+, C3++	IIIb	1/25	4
2	IgA+++, C3++	IIb	0/11	2
3	IgG+, IgA+++, IgM+-, C3+++	IIb	0/9	4
4	IgG+, IgA++, C3++	IIIb	1/13	5
5	IgA++, C3++	IIb	0/30	1
6	IgA+++, IgM+, C3++	IIb	0/17	5
7	IgG+, IgA+++, C3++	IIb	0/21	4
8	IgG+, IgA+++, IgM+, C3+	IIb	0/5	2
9	IgA++	IIb	0/25	4
10	IgG+-, IgA++, C3 +	IIb	0/31	4
11	IgG+, IgA+++, C3++	IIb	0/18	4

**Table 3:** Comparison of clinical and pathological presentation between DEP- HSPN (class IIb) and non-DEP-HSPN (class IIb).

	DEP-HSPN (n=9)	Non-DEP-HSPN (n=43)	*p* value		
Age (years)	9.57±0.81	9.44±0.40	0.896		
Gender (M/F)	5/4	31/12	0.328		
Abdominal pain	3/9	15/43	0.929		
Arthralgia	4/9	19/43	0.989		
Simplified proteinuria	0/9	1/43	0.644		
Hematuria & proteinuria	1/9	26/43	**0.007**		
Acute nephritis	1/9	2/43	0.450		
Nephrotic syndrome	7/9	14/43	**0.012**		
Serum IgA (g/L)	1.47±0.59	1.88±0.72	0.119		
Serum C3 (g/L)	1.22±0.17	1.18±0.30	0.707		
IgA deposits	3/9	12/43	0.744		
IgA+IgG deposits	3/9	7/43	0.238		
IgA+IgM deposits	1/9	13/43	0.240		
IgA+IgM+IgG deposits	2/9	11/43	0.832		

Simplified proteinuria: urine protein >4 mg/kg/d, but <50 mg/kg/d.

Hematuria and proteinuria: > 5 RBCs/HPF with urine protein >4 mg/kg/d, but <50 mg/kg/d.

Acute nephritis: hematuria plus at least one of the following: increased serum creatinine, hypertension, or oliguria.

Nephrotic syndrome: urine protein >50 mg/kg/d, serum albumin <2.5 g/dL, plus edema and hyperlipidemia.

**Table 4:** Treatment and outcome.

*Case*	*MP Pulse Therapy*	*Prednisone*	*Others*	*Proteinuria (month)*	*Outcome*
1		+	MMF	7	RBC, 14/HP after 15 months
2			Tripterygium	2	Normal urine test after 13 months
3		+	MMF	4	RBC, 14/HP after 20 months
4	+	+	CTX	5	Normal urine test after 17 months
5		+	Tripterygium	1	Normal urine test after 7 months
6	+	+	CTX	5	RBC, 10/HP after 15 months
7		+		4	RBC, 8/HP after 19 months
8		+	Tripterygium	2	RBC, 6/HP after 17 months
9			Tripterygium	4	Normal urine test after 13 months
10	+	+	CTX	4	Normal urine test after 15 months
11		+	MMF	4	RBC, 15/HP after 13 months
					

HP: hypertension; MP: methylprednisolone; PN: prednisone; MMF: mycophenolate mofetil; CTX: cyclophosphamide; RBC: red blood cell; HPF: high-power field.
